# Preparation of Self-Assembled Nanoparticle–Polymer Hybrids from Modified Silica Nanoparticles and Polystyrene-Block-Polyacrylic Acid Vesicles via the Co-Precipitation Method

**DOI:** 10.3390/polym15020444

**Published:** 2023-01-14

**Authors:** Jil Mann, Georg Garnweitner, Carsten Schilde

**Affiliations:** 1 Institute for Particle Technology, Technische Universität Braunschweig, Volkmaroder Str. 5, 38104 Braunschweig, Germany; 2 Laboratory for Emerging Nanometrology, Technische Universität Braunschweig, Langer Kamp 6A, 38106 Braunschweig, Germany

**Keywords:** nanoparticle–polymer hybrid, synthesis, modification, block copolymer, self-assembly, encapsulation, co-precipitation, vesicle, fusion

## Abstract

Nanoparticle–polymer hybrids are becoming increasingly important because seemingly contrasting properties, such as mechanical stability and high elasticity, can be combined into one material. In particular, hybrids made of self-assembled polymers are of growing interest since they exhibit high structural precision and diversity and the subsequent reorganization of the nanoparticles is possible. In this work, we show, for the first time, how hybrids of silica nanoparticles and self-assembled vesicles of polystyrene-block-polyacrylic acid can be prepared using the simple and inexpensive method of co-precipitation, highlighting in particular the challenges of using silica instead of other previously well-researched materials, such as gold. The aim was to investigate the influence of the type of modification and the particle size of the silica nanoparticles on the encapsulation and structure of the polymer vesicles. For this purpose, we first needed to adjust the surface properties of the nanoparticles, which we achieved with a two-step modification procedure using APTES and carboxylic acids of different chain lengths. We found that silica nanoparticles modified only with APTES could be successfully encapsulated, while those modified with APTES and decanoic acid resulted in vesicle agglomeration and poor encapsulation due to their strong hydrophobicity. In contrast, no negative effects were observed when different particle sizes (20 nm and 45 nm) were examined.

## 1. Introduction

Self-organized systems are the basis of intelligent life. A well-known example is our cell membrane, which is precisely constructed from self-assembled phospholipids [1]. Therefore, understanding and controlling self-assembly mechanisms open up many new possibilities, such as the development of functional nanosystems that require a high degree of structural precision. Recently, hybrid structures of self-assembled organic materials with embedded inorganic nanoscale components have become the focus of research because various advantageous properties can be combined in a single material [1,2,3]. Furthermore, it is possible to achieve a high structural diversity and precise arrangement of nanoparticles [3,4,5,6] to protect the embedded nanoparticles from unwanted interactions, such as agglomeration or degradation, with the surrounding medium [4,5]; to achieve controlled release of the nanoparticles [7]; or to increase thermal and mechanical resistance [8,9]. These nanoparticle–polymer hybrids are of interest for many potential applications, for example, in nanoreactors [10], drug delivery systems [11,12,13,14], sensors [15], smart materials with stimuli-responsive properties (such as thermo-responsive [16,17], light-responsive, [18] and magnetic-responsive [19,20,21] properties), water treatment [22], hybrid solar cells [23], and intrinsic self-healing materials [24].

Many amphiphilic molecules, such as the mentioned phospholipids, are able to self-assemble into hierarchical structures. Block copolymers were extensively investigated since they have longer phase transformation times due to their high molecular weights in comparison to low-molecular-weight amphiphiles. This allows, on the one hand, better control over the forming mesoscopic structures and, on the other hand, deeper insights into inter- and intramolecular reorganization processes because the metastable transition morphologies exist in a larger time window and can, thus, be studied more easily [25,26,27,28,29].

One strategy for triggering the self-assembly of block copolymers is the co-solvent method. The block copolymer is first dissolved in a solvent that is sufficient for both the hydrophobic and the hydrophilic parts of the molecule (e.g., dioxane or tetrahydrofuran). Subsequent addition of a selective solvent (e.g., water), in which one part of the polymer is insoluble, leads to energetically unfavorable interactions, causing the molecules to self-assemble with the aim of interfacial minimization. This process is also referred to as microphase separation since one part of the block copolymer stays in the dissolved form [1]. The resulting morphology of the polymer structure depends on the packing parameter p, first described by Israelachvili et al. [30] in 1976. It describes the ratio of the volume of the hydrophobic part of the amphiphilic molecule to the contact area of the hydrophilic part and the length of the hydrophobic polymer chain. The best-known morphologies are spherical and cylindrical micelles, lamellar structures, and vesicles [1,29,30,31,32,33]. However, in recent years, many other morphologies, such as toroidal micelles, Janus vesicles, sunflower-like vesicles, and helical structures, have been reported [34,35,36,37,38,39,40,41,42,43].

**Figure 1:**
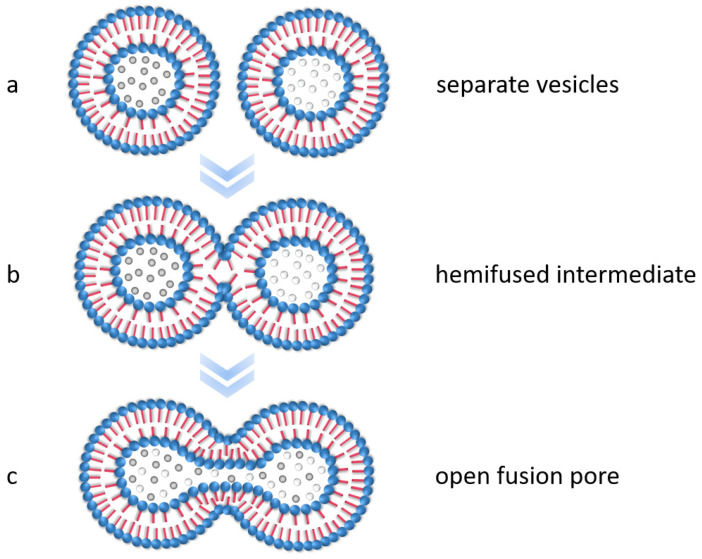
Schematic representation of the basic processes in membrane fusion. (**a**) Before the membranes come in contact, (**b**) after the outer membranes come in contact and fuse to form a hemifused intermediate, and (**c**) after the fusion pores open through reorganization processes. Figure inspired by [51].

Many different factors determine the morphology, such as the type and concentration of the solvents [44] and the block copolymer [45,46], temperature, and the presence of additives [6,46,47]. However, often, the thermodynamically stable structure is not immediately available after microphase separation but forms in the further course of the process, with metastable transition morphologies as intermediate stages. In this context, it was postulated that among other mechanisms, fusion processes between the structures play a decisive role [35,48,49]. Although the fusion of membranes depends on the system (e.g., the type and size of the vesicle), it occurs in specific steps, shown schematically in Figure 1 [50,51]. First, the membrane surfaces must be brought in contact, which leads to a localized impairment of the double layer, reorganization, and the fusion of the outer membranes. This hemifused state (often the so-called stalk-like state) is an intermediate product. Over time, rearrangement mechanisms lead to the formation and opening of a fusion pore until the contents of the two vesicles have mixed and, finally, a single large vesicle is formed [51,52].

To generate nanoparticle–polymer hybrids, a suitable method is required for encapsulating the nanoparticles. Wang et al. [53] provided a detailed overview of different encapsulation methods for creating self-assembled block-copolymer-based structures. One is the co-precipitation method, which is triggered by either solvent evaporation or solvent exchange and is the most preferred method for preparing hybrid materials from self-assembled amphiphiles [54]. The focus of this article is solvent exchange, where a homogeneous solution of stabilized nanoparticles and the block copolymer in a water-miscible organic solvent (e.g., tetrahydrofuran) is mixed with a selective solvent, such as water. Both the nanoparticles and the insoluble part of the polymer agglomerate, whereas the water-soluble part of the polymer remains in solution. This results in non-covalent attachment of the nanoparticles within the mesoscopic structure [55,56]. This method is simple, fast, and economical as it usually requires few chemicals, no complex design, and little energy [57]. The disadvantage of this method, however, is that the particles must be sufficiently stabilized in the solution to avoid aggregation and, thus, an inhomogeneous product [58]. Another method of encapsulating nanoparticles into a polymeric matrix uses interfacial instabilities of emulsion droplets. Unlike the method before, the nanoparticles and the block copolymer are dissolved in a water-insoluble organic solvent (for example, chloroform) and then dispersed in water. The extraction of the organic solvent shrinks the emulsion droplets until interfacial instabilities result and new interfaces are formed, leading to the formation of micellar aggregates with encapsulated nanoparticles in the water [59]. With the heating–cooling method, both block copolymers with long hydrophobic blocks and those with long hydrophilic blocks can be used. Here, a solution of block copolymer and stabilized nanoparticles is first heated to 110 °C for 2 h and then slowly cooled. The critical micelle concentration (CMC) of the polymer slowly decreases with temperature until the block copolymers and nanoparticles assemble into hybrids [4]. Another method of preparing nanoparticle–polymer hybrids is the use of electrostatic interactions. The main advantage of this strategy is that the nanoparticles can be precisely localized both in the micelle core and at the interface. Here, first, microphase separation generates micellar structures. Then, oppositely charged nanoparticles (for example, positively charged gold nanoparticles) absorb into the corresponding microdomain of the block copolymer structure (for example, within the areas consisting of negatively charged polyacrylic acid groups) [49]. Solvent-induced self-assembly by, e.g., non-covalent interaction has also been applied to prepare nanoparticle–polymer hybrids [21,60]. For example, Meng et al. [21] used this method to prepare self-assembled core–shell composite nanofibers with magnetic Fe_3_O_4_ nanoparticles and conjugated block copolymers using orthogonal non-covalent interactions. For this purpose, first, the polymer was dissolved in chlorobenzene. Then, a defined amount of acetone was added under stirring and the mixture stirred for 9 h at room temperature. Then, the magnetic nanoparticles were added to the polymer nanofiber solution and stirring continued for another hour, resulting in a nanofiber hybrid solution. Finally, this solution could be directly used for solar cell fabrication by diluting it 100 times with a solvent mixture of chlorobenzene and acetone. Another method for creating nanoparticle–polymer hybrids is the templated self-assembly method. Here, the interface between air and water is exploited for the self-assembly of amphiphilic molecules. By evaporating the solvent from a mixture of nanoparticles and block copolymers on a substrate, defined hybrid structures can be generated [61,62].

As diverse as the encapsulation methods are the types of embedded nanoparticles. Some well-studied nanomaterials so far are gold, silver, iron oxide, and aluminum oxide nanoparticles, which are primarily used because of their properties, such as magnetism [17,63,64,65,66]. To the best of our knowledge, there is currently no publication in which silica nanoparticles are encapsulated in self-assembled mesoscopic block copolymer structures such as vesicles using the co-precipitation method applied here. Silica is a promising material for preparing nanoparticle–polymer hybrids due to its low toxicity, good availability, and versatile applications, e.g., for increasing the mechanical hardness [9,67,68,69,70,71,72]. However, a disadvantage of silica is that it does not have magnetic properties, such as iron nanoparticles, so applications in the field of controlled release by magnetic heating are not possible without further modification measures [17].

The sol–gel method is a common process for preparing silica–polymer hybrids [73,74,75,76,77,78,79]. The molecular precursor forms a three-dimensional network within the polymer matrix during the sol–gel reaction [78]. It is a simple and inexpensive method to produce hybrid materials that can be used in a wide range of applications, such as catalysis, drug release, and separation technology [80,81,82,83]. However, the silica particles form covalent bonds with each other [77,79] or with the polymer matrix [84] and the polymer material is often not capable of forming self-assembled hierarchical structures [73,74], resulting in a non-dynamic system. Subsequent reorganization of the encapsulated nanoparticles by the selective induction of phase transformation processes of the mesoscopic polymer structures, as shown by Wang et al. [3], with gold nanoparticles encapsulated in PS-b-PAA cylinders, would not be possible. The main advantage of dynamic nanoparticle–polymer hybrids is that structural defects caused during nanoparticle assembly can be subsequently repaired [3].

Compared to the hybrid systems of self-assembled polymers known from the literature so far, the use of silica leads to completely different challenges with regard to encapsulation, requiring the development of a new process chain. Due to its similar surface chemistry, silica serves as a model particle system for metal oxides. For example, Kockmann et al. [65,72,85] have already shown that metal oxide nanoparticles of ZrO_2_ and Al_2_O_3_ can be hydrophobized and incorporated into a polymer matrix using the functionalization method applied here. Thus, it is expected that the findings reported in this study are transferable to other metal oxide systems. The materials shown so far, such as gold, are hydrophobized and simultaneously stabilized by adding a hydrophobic modifier with a thiol group in the molecule to the gold suspension before precipitation [3,4,55]. Since silica is not a metal and, thus, does not have a high affinity for thiol groups, another method of hydrophobization must be used and be examined and assessed with regard to encapsulation. One possibility to hydrophobize the nanoparticles is to bond APTES and carboxylic acids to the particle surface. The advantage of this method is that different hydrophobicities can be achieved, which can significantly influence the encapsulation and structure formation of the polymers, as seen in Section 3.2. The disadvantage, however, is that suspension instabilities occur, especially with increasing chain length.

**Figure 2:**
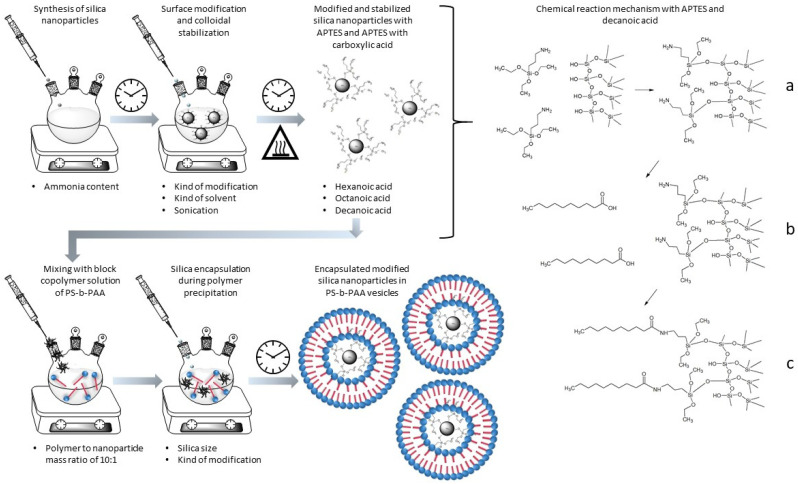
Schematic representation of the process for the production of the self-assembled nanoparticle–polymer hybrids from silica and PS-b-PAA vesicles, involving synthesis, surface modification, colloidal stabilization, and encapsulation with the co-precipitation method as well as the illustration of the reaction mechanism of the two-stage modification method using the example with APTES and decanoic acid. (**a**) First modification step by chemically coupling APTES to the silica surface, (**b**) addition of decanoic acid to the APTES-modified silica nanoparticles, and (**c**) second modification step by chemically coupling decanoic acid to the silica–APTES surface.

Therefore, the aim of this study is to show, for the first time, how the simple and economical method of co-precipitation can be used to prepare silica–polymer hybrids of self-assembled PS-b-PAA vesicles, which exhibit high dynamics due to their non-covalent bindings and allow the possibility of subsequent nanoparticle reorganization. In particular, the influence of factors such as nanoparticle size and type of modification of the nanoparticles on the mesoscopic structure will be elucidated. For this purpose, the characterization of the nanoparticles and the adjustment of their surface properties are important prerequisites. Figure 2 shows the established process chain from the synthesis and modification of the silica nanoparticles to encapsulation in PS-b-PAA vesicles using the co-precipitation method.

## 2. Materials and Methods

### 2.1. Materials

Polystyrene-block-polyacrylic acid (PS-b-PAA, batch number: MKBQ5839V) was purchased from Sigma Aldrich (Steinheim, Germany). The molecular weights were determined by GPC with the solvent THF and PS as the standard. A value of 27,617 g/mol was obtained for PS and 1761 g/mol for PAA, leading to a total molecular weight of 29,378 g/mol with a polydispersity ≤1.1. The degree of polymerization of PS was 275 and of PAA 30. Deionized water was used as the selective solvent for polystyrene and for the silica synthesis. Tetrahydrofuran (THF, not stabilized, for HPLC) from Sigma Aldrich (Steinheim, Germany) served as the solvent for PS-b-PAA. Tetraethylorthosilicate (TEOS, for synthesis; purity ≥ 99.0%), aqueous ammonia solution 25% (for analysis, Reag. Ph. Eur.), 3-aminopropyl triethoxysilane (APTES; purity ≥ 98.0%), hexanoic acid (for synthesis; purity ≥ 98.0%), octanoic acid (for synthesis; purity ≥ 99.0%), decanoic acid (for synthesis; purity ≥ 98.0%), N,N’-diisopropylcarbodiimide (DIC, for synthesis; purity ≥ 99.0%) from Sigma Aldrich (Steinheim, Germany), and absolute ethanol (purity ≥ 99.8%, Reag. Ph. Eur.) from VWR chemicals (Darmstadt, Germany) were used for silica synthesis and surface functionalization.

### 2.2. Silica Synthesis, Modification, and Colloidal Stabilization

Stöber synthesis was used to prepare silica particles (SiO_2_) [86]. Ethanol (2.55 mol), deionized water (1.05 mol), and ammonia (0.04, 0.06, 0.10, 0.26, 0.52, 0.63, 0.79, and 1.05 mol) were mixed completely under stirring for 15 min. The entire amount of the TEOS precursor (0.11 mol) was added rapidly, initiating sol–gel synthesis. After 24 h, with continuous stirring at 400 rpm, the particles were completely grown. Finally, the silica particles were separated from the water and ammonia and transferred to pure ethanol (absolute). For this purpose, the suspensions were centrifuged at 8500 rpm for 25 min and redispersed in ethanol. This procedure was repeated 3 times. Suspensions with particles smaller than 70 nm could no longer be efficiently centrifuged due to their size. Here, the particles could be transferred to ethanol using a rotary evaporator. Ammonia and water were removed here under an elevated temperature (40 °C) and reduced pressure (down to 70 mbar, just below the vapor pressure of water at 40 °C), and the evaporated amount was replenished with ethanol.

The method for modifying silica particles was derived from Kockmann et al. [72]. 

First, silica particles were modified with APTES, choosing a molar ratio of silica to APTES of 1:1. Both the APTES in ethanol solution and the silica suspension were preheated in separate flasks in an oil bath to about 50 °C before the silica suspension was added dropwise with vigorous stirring to the APTES solution. The flask was sealed airtight, and the mixture was stirred at 78 °C for at least 12 h under stirring at 400 rpm. Then, the particles were centrifuged a total of three times at 8000 rpm for 15 min and redispersed in ethanol. Applying the ninhydrin test [72], the number of washing steps required to remove all free APTES in the supernatant was determined in advance. 

Second, carboxylic acids with different chain lengths (hexanoic, octanoic, and decanoic acid) were coupled to the amine groups present on the particle surface. The molar ratio of carboxylic acid to silica (as SiO_2_) was 1:1. A coupling reagent was needed to support the reaction. In this case, diisopropylcarbodiimide (DIC) was used in a molar ratio of 3:2 to the respective carboxylic acid. For this purpose, first, the carboxylic acid was dissolved in ethanol and heated to about 50 °C in a flask. At the same time, the suspension with APTES-modified silica nanoparticles was heated to 50 °C in a flask and then added dropwise to the carboxylic acid solution. After stirring at 78 °C for 12 h, the particles were washed. For this purpose, the suspensions were centrifuged at 8500 rpm for 25 min and redispersed in ethanol. This procedure was repeated 3 times.

### 2.3. Encapsulation of Modified Silica in PS-b-PAA Vesicles

The modified silica nanoparticles were finally encapsulated in PS-b-PAA vesicles. Here, we initially used the silica nanoparticles modified with APTES and with APTES–decanoic acid, the two types of modification that differ most clearly from one another due to their hydrophobicity. The experimental conditions, with regard to the type and amount of solvent, polymer concentration, temperature, stirring speed, and duration, were determined on the basis of experience from previous investigations. For encapsulation, the polymer was first dissolved in THF, which is a good solvent for both polystyrene and polyacrylic acid, and the mixture stirred for at least 12 h. The initial concentration of the polymer solution was 3 mg/mL, and the polymer–nanoparticle mass ratio in the product amounted to 10:1, a ratio that seemed to provide enough nanoparticles for encapsulation but not so many nanoparticles that the structure formation of the vesicles was disturbed during precipitation. The SiO_2_–APTES suspension was then treated with an ultrasonic sonotrode (Bandelin Sonopuls mini20, Berlin, Germany) for 2 min before it was added to the polymer solution with vigorous stirring. SiO_2_–APTES–decanoic acid was first transferred to THF before being added to the polymer solution. The mixture was then stirred at 400 rpm for about 30 min to ensure homogeneous mixing. In all experiments, a clear/slightly opaque solution was obtained with no agglomerates or sediments, indicating good stability of the polymer–nanoparticle dispersion before precipitation. Water was then added to the polymer solution as a selective solvent at a rate of 25 μL/min under a stirring speed of 400 rpm until a water content of 14 wt% was reached. At this point, the product volume was 5 mL and the polymer concentration was 0.1 wt%. The solution was then stirred at 100 rpm for 3 h to obtain the polymer vesicles. The morphology was assessed after 24 h of the process time to ensure that the vesicles in this case represented the thermodynamic equilibrium structure. To freeze the morphology after the end of the process, the selective solvent water was added to the mixture (the solvent was three times the total amount of the mixture). Finally, the vesicles were washed with water by centrifuging the solution at 9500 rpm for 15 min and redispersing in water. In our experience, no changes in the morphology of the polymer vesicles resulted from this purification procedure. The TEM and SEM samples were prepared immediately afterward.

### 2.4. Characterization of Particle Size and Surface Modification

Particle sizes were determined using dynamic light scattering (instrument: Zetasizer from Malvern, Worcestershire, UK) with a quartz glass cuvette (Hellma Analytics, Type 100 QS, Müllheim, Germany). Per sample, 3 measurements were performed. To investigate the influence of sonication on particle size and distribution, the samples were additionally treated with an ultrasonic sonotrode for 2 min before the measurement.

To yield a dry powder, about 2 mL of the suspension was first stored in a vial at 80 °C for 2 h in a muffle oven and then stored in a vacuum desiccator for at least 3 days. A few milligrams were sufficient to enable Fourier-transform infrared spectroscopy (FTIR) and thermogravimetric analysis (TGA). For the TGA measurements, the powder samples were heated to 700 °C under atmospheric conditions and the mass loss was determined.

### 2.5. Encapsulation Imaging

Samples were visually assessed using a transmission electron microscope (TEM, instrument: Tecnai G2 F20 TMP from FEI, voltage: 200 kV, Frankfurt am Main, Germany) and a scanning electron microscope (SEM, instrument: Helios G4 CX from FEI, voltage: 5 kV, Frankfurt am Main, Germany). Between 50 µL and 150 µL of the suspensions were dropped onto a copper grid for TEM measurements and onto a membrane filter (Whatman^®^ Nuclepore™ Track–etched polycarbonate membranes with pore diameter of 0.2 µm, Dassel, Germany) and dried in atmospheric air. The membrane filters for the SEM measurements were then glued to a copper grid and sputtered with 4 nm platinum.

## 3. Results and Discussion

In this section, the process chain for preparing nanoparticle–polymer hybrids from silica nanoparticles and PS-b-PAA vesicles will be shown and discussed analogously to Figure 2. For this purpose, silica synthesis will be demonstrated first, followed by the modification of the particles to ensure optimal surface properties or hydrophobicities of the particles with regard to encapsulation. Next, we will discuss the challenges in the modification with respect to re-agglomeration and stabilization of the particles. Finally, the encapsulation of the modified silica particles in PS-b-PAA vesicles will be presented.

### 3.1. Silica Synthesis, Modification, and Colloidal Stabilization

In this section, we will (1) show the synthesis of silica particles by the Stöber method, (2) show how the synthesized silica particles are modified with APTES and carboxylic acids of different chain lengths (hexanoic, octanoic, and decanoic acids), (3) use FTIR and TGA measurements to evaluate the process, (4) investigate the suspension stabilities of the modified silica nanoparticles by DLS measurements, and (5) demonstrate and discuss different re-agglomeration methods (ultrasonic and solvent exchange). 

**Figure 3:**
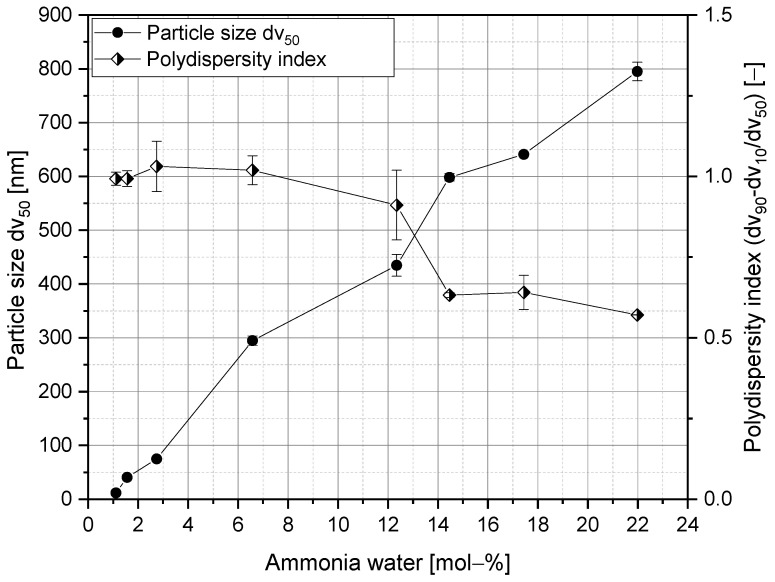
Particle size and polydispersity index of the silica nanoparticles by varying the amount of ammonia when using the Stöber method.

To begin with, it was crucial to select a simple and reproducible method for silica synthesis. By 1968, Stöber et al. [86] had discovered that particle sizes and size distributions of silica nanoparticles depend on the synthesis conditions (e.g., by varying the ammonia content). Over time, numerous other researchers have confirmed this [68,87,88,89]. Therefore, we chose the Stöber method to synthesize the nanoparticles and evaluated the exact conditions for generating a targeted particle size by successively increasing the amount of ammonia and measuring the particle size (as the volume mean diameter dv_50_). As can be seen in Figure 3, the particle size of silica particles can be controlled between 12 nm ± 0.08 nm (at 1 mol–% ammonia, related to the total molarity in the synthesis mixture) and about 800 nm ± 17.28 nm (at 22 mol–% ammonia).

At lower or higher concentrations, homogeneous suspensions could no longer be observed. Bogush et al. [68] reached a similar conclusion. Under the same conditions, they were able to synthesize particles between 20 nm and 800 nm. However, for our study, we selected particle sizes smaller than 50 nm (concrete: 45 nm ± 1.88 nm and 20 nm ± 8 nm) to ensure that the particles could be integrated into the polymer vesicles.

For encapsulation, it is also critical to ensure high affinity between the nanoparticle and the block copolymer. It was postulated that the surface properties of the silica particles featuring hydroxyl groups might hinder successful encapsulation. Therefore, the surface properties were specifically varied using different modifiers. The modification was performed in a two-step process: (1) the particle surface is modified with APTES and (2) the particle surface is modified with a carboxylic acid (hexane, octane, and decanoic acid). The main advantage of this two-step process is that carboxylic acid residues of any length can be coupled to the surface, resulting in different degrees of hydrophobicity. These can be crucial for the success of the process when preparing nanoparticle–polymer hybrids, which will be demonstrated in Section 3.2.

**Figure 4:**
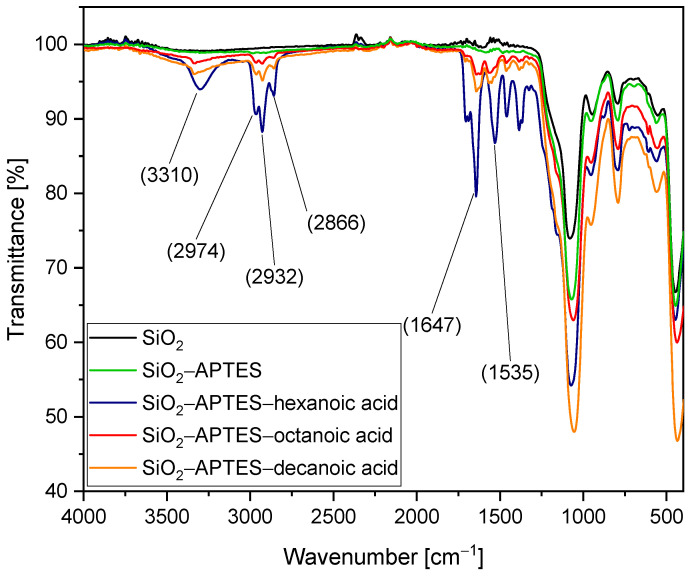
FTIR spectra of 45 nm silica nanoparticles: unmodified silica particles used as a reference (SiO_2_); SiO_2_ modified with APTES (SiO_2_–APTES); and SiO_2_ modified with APTES with hexanoic (SiO_2_–APTES–hexanoic acid), octanoic (SiO_2_–APTES–octanoic acid), and decanoic acid (SiO_2_–APTES–decanoic acid).

We verified our method by performing FTIR measurements on 45 nm unmodified and modified silica particles. See Figure 4. Transmission values larger than 1 are exclusively due to measurement artifacts and can, therefore, be neglected. The minima in the lower wavelength range (approx. 450 to 1000 cm^−1^) are characteristic of silica (fingerprint range) and therefore occur in all spectra. The characteristic vibrations of the organics present in the modified particles (APTES and APTES with hexanoic, octanoic, and decanoic acid) can be seen in the wavelength range between just above 1000 cm^−1^ and 3400 cm^−1^. However, we will not discuss each individual minimum in detail here as the successful application of this coupling strategy to silica particles has already been demonstrated before [62]. First, it can be seen that the spectrum of the particles modified with APTES is highly similar to the one of pure silica used as a reference. Compared to the FTIR spectrum of APTES-modified ZrO_2_ nanoparticles [85], the expected signals of the CH_2_ and CH_3_ groups are only slightly visible, which we attribute to the larger size of the silica nanoparticles resulting in a much lower organic content for similar density at the particle surface. However, after additional coupling of hexanoic, octanoic, and decanoic acid, the expected transmission bands are clearly visible.

The minimum at 3310 cm^−1^ reveals the presence of secondary amines (R–NH–R), mainly characterized by NH stretching vibration. In contrast to primary amines, which show a double minimum in the range between 3310 and 3350 cm^−1^, secondary amines here show only one. Furthermore, the characteristic hydrocarbon vibrations of carboxylic acids can be observed in the wavelength range between 2840 and 3000 cm^−1^. Of particular interest are the wavelength ranges between 1597 and 1672 cm^−1^ as well as between 1480 and 1575 cm^−1^. Amide I vibrations, mainly caused by C=O stretching vibrations, could be detected in the first mentioned range and amide II vibrations, dominated by NH bending vibrations, could be detected in the second range [90]. The minima shown in this FTIR spectrum thus indicate that APTES and the respective carboxylic acid are covalently linked and bound to the particles. First, the APTES molecules are linked to the surface of the silica particles with the cleavage of ethanol, resulting in the formation of covalent Si–O–Si bonds. Next, amidation with the carboxylic acid takes place. Under cleavage of water, the carboxylic acid is covalently bound to the APTES-modified silica surface. The characteristic CO–NH group is formed, which can be detected by FTIR. The hexanoic acid–modified particles exhibit noticeably higher signal intensity for the expected organic vibrations in the range between 1000 cm^−1^ and 3400 cm^−1^ compared to the other samples, attributed to a larger amount of sample measured, as well as a higher organic content, which might be explained by some variation in the APTES content due to experimental variations.

**Figure 5:**
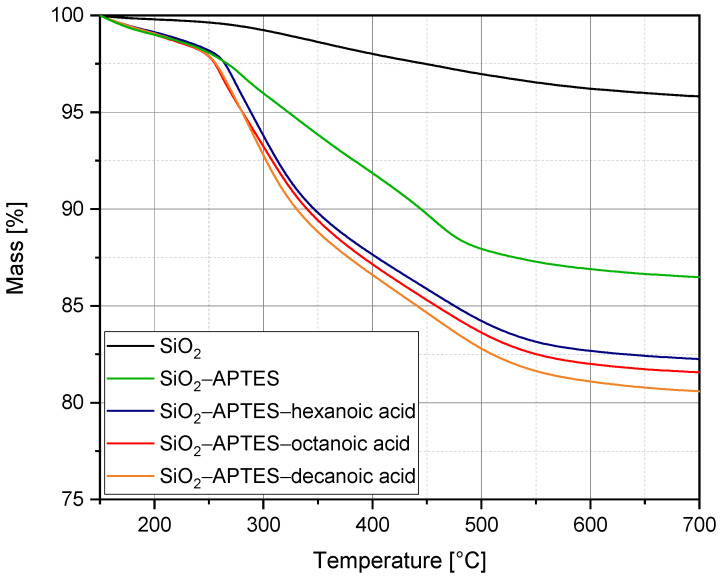
Thermogravimetric analysis of unmodified silica nanoparticles (SiO_2_) as a reference and silica nanoparticles after modification with APTES (SiO_2_–APTES) as well as after two-step modification with APTES and different carboxylic acids (SiO_2_–APTES–hexanoic acid, SiO_2_–APTES–octanoic acid, and SiO_2_–APTES–decanoic acid).

To further characterize the modified silica nanoparticles, we performed thermogravimetric analysis. Figure 5 illustrates the change in mass (in %) versus temperature. The starting temperature was set at 150 °C because up to this temperature, only adsorbed water is removed from the powder samples [91]. The silica reference shows a significantly lower weight loss, of only 4.2%, compared with the modified particles. All other curves exhibit a slower drop up to about 250 °C, followed by a marked drop up to about 500 °C, and a much less marked drop up to 700 °C. This is similar for all modifications, although the longer the chain of the modifier or the higher the molar mass bound to the silica particles, the more pronounced the drop between 250 °C and 500 °C. Therefore, it can be deduced that mainly the modifier is decomposed in this temperature interval. Overall, we could detect a mass loss of 13.5% for the particles modified with APTES, of 17.7% for the particles modified with APTES–hexanoic acid, of 18.4% for the particles modified with APTES–octanoic acid, and of 19.4% for the particles modified with APTES–decanoic acid. Accordingly, analogous to other literature sources [65,91,92], a trend emerged that the higher the molecular weight, the higher the mass loss. In our specific case, it was even possible to determine a direct linear relationship between the molecular weight of the modifier and the mass loss.

**Figure 6:**
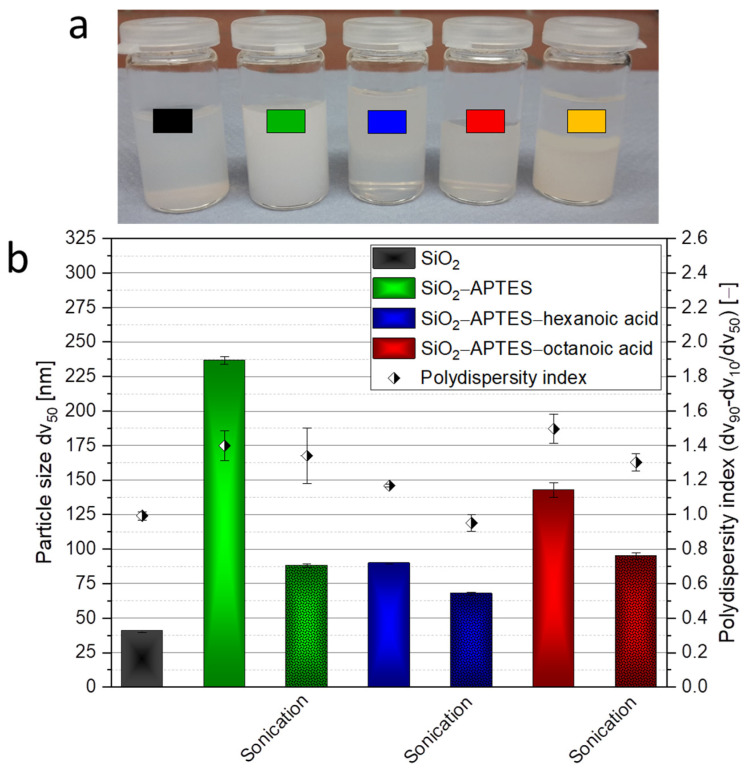
Photograph of silica suspensions (**a**) as well as DLS measurements of the particle size and particle size distributions of the unmodified and modified silica nanoparticles (**b**). (**a**) Suspensions in the solvent ethanol (black: SiO_2_ as a reference; green: SiO_2_–APTES; blue: SiO_2_–APTES–hexanoic acid; red: SiO_2_–APTES–octanoic acid; orange: SiO_2_–APTES–decanoic acid). (**b**) SiO_2_ as a reference and modified particles (SiO_2_–APTES, SiO_2_–APTES–hexanoic acid, and SiO_2_–APTES–octanoic acid) in ethanol with and without sonication. SiO_2_–APTES–decanoic acid could not be measured due to strong sedimentation.

The silica particles had to be modified so they could be encapsulated, but this changed the behavior of the particles in the suspension. Figure 6a shows a photograph of the silica suspensions in ethanol (black: SiO_2_ as a reference; green: SiO_2_–APTES; blue: SiO_2_–APTES–hexanoic acid; red: SiO_2_–APTES–octanoic acid; orange: SiO_2_–APTES–decanoic acid). Figure 6b presents the DLS results. The particle size and polydispersity (PDI) of the modified silica particles are increased compared with the unmodified particles. This was most noticeable for the particles modified with APTES (237 nm ± 2.62 nm with a PDI of 1.4 ± 0.08). In contrast, the particles modified with APTES–hexanoic acid showed only a slight increase in particle size (90 nm ± 0.41 nm with a PDI of 1.17 ± 0.008) and the particles modified with APTES–octanoic acid demonstrated slightly larger particle sizes and particle size distribution (143 nm ± 5.25 nm with a PDI of 1.5 ± 0.08), although both were significantly lower than the size and distribution after modification with APTES only. Presumably, the hydrocarbon groups of the carboxylic acids had a higher compatibility with the solvent ethanol than APTES with its amino group, which is why the particles modified with APTES have a higher tendency to agglomerate.

The encapsulation of larger agglomerates could lead to inhomogeneous hybrids as well as disrupt the structural formation of the polymer vesicles. Hence, efforts were made to re-agglomerate them again using ultrasound. This showed good success for the particles modified with APTES (88 nm ± 1.14 nm with a PDI of 1.34 ± 0.16), with APTES and hexanoic acid (68 nm ± 0.97 nm with a PDI of 0.95 ± 0.05), and with APTES and octanoic acid (95 nm ± 2.40 nm with a PDI of 1.3 ± 0.05). Ultrasound had to be applied to the suspensions directly before encapsulation. Visual observations also showed clear differences between the modified particles. As seen in Figure 6a, compared with the unmodified silica particles, the suspension with APTES-modified silica particles became cloudy because of agglomeration. The suspensions with APTES–hexanoic acid– and APTES–octanoic acid–modified particles, however, were less cloudy and did not show any sedimentation. In contrast, the suspension with APTES–decanoic acid–modified silica particles exhibited a clear phase separation with sedimented agglomerates and ultrasound could not contribute to satisfactory re-agglomeration. In this case, DLS measurements were not recorded because the agglomerates were outside the instrument’s measurement range of 1 µm.

**Figure 7:**
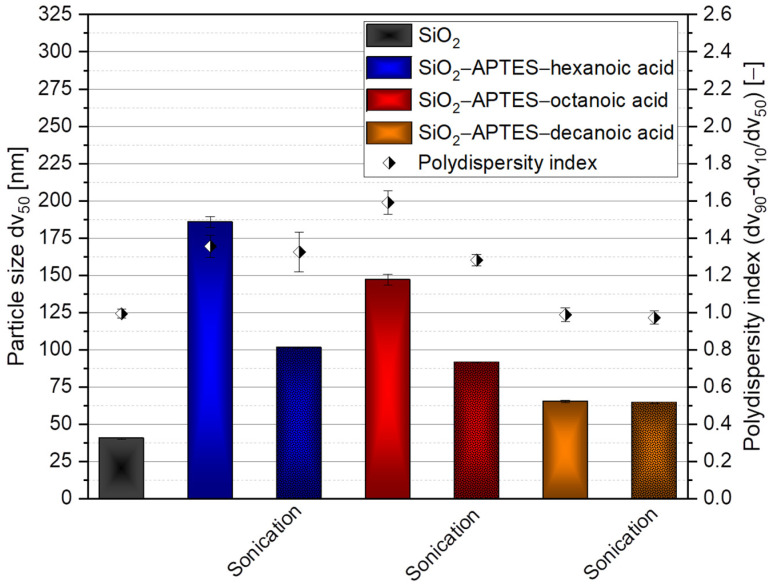
DLS measurements of unmodified silica as a reference (SiO_2_) in ethanol, SiO_2_–APTES–hexanoic acid, SiO_2_–APTES–octanoic acid, and SiO_2_–APTES–decanoic acid in the solvent THF with and without sonication.

Therefore, another method was established. We transferred the silica particles modified with APTES–decanoic acid and, for comparison, the particles modified with APTES–hexanoic acid and APTES–octanoic acid to THF and measured the particle size and the particle size distribution (see Figure 7). As expected, solvent change seemed to be an effective method for stabilizing the hydrophobic particles. Comparing the n–octanol/water distribution coefficients of ethanol with −0.35 [93] and THF with 0.45 [94], a difference in hydrophobicity was observed that appeared to be sufficient to re-agglomerate the APTES–decanoic acid–modified particles (65 nm ± 0.90 nm with a PDI of 0.99 ± 0.04). Sonication showed no significant enhancement in particle size or distribution (64 nm ± 0.31 nm with a PDI of 0.97 ± 0.04), so it was assumed that this was the best possible re-agglomeration result. As already mentioned, the particles modified with APTES and hexanoic acid (186 nm ± 3.68 nm with a PDI of 1.35 ± 0.06) as well as APTES and octanoic acid (147 nm ± 3.74 nm with a PDI of 1.59 ± 0.06) were also transferred to THF and we studied the effect on particle size and particle distribution. We found that the shorter the carbon chain and, thus, the more hydrophilic the particle surface, the larger the particles or agglomerates as the interaction forces between the particles and the solvent decreased. The particles modified with APTES–octanoic acid did not exhibit any significant difference for the two solvents, as they possess moderate hydrophobicity. Similar to the suspensions with ethanol (compare Figure 6), we also found that treatment with ultrasound can have a positive effect on re-agglomeration (especially for APTES–hexanoic acid (101 nm ± 0.47 nm with a PDI of 1.32) and APTES–octanoic acid (91 nm ± 0.23 with a PDI of 1.28 ± 0.03)).

### 3.2. Encapsulation of Modified Silica in PS-b-PAA Vesicles

After the silica nanoparticles were successfully modified and stabilized, they had to be encapsulated in PS-b-PAA vesicles, which we achieved using the co-precipitation process. 

**Figure 8:**
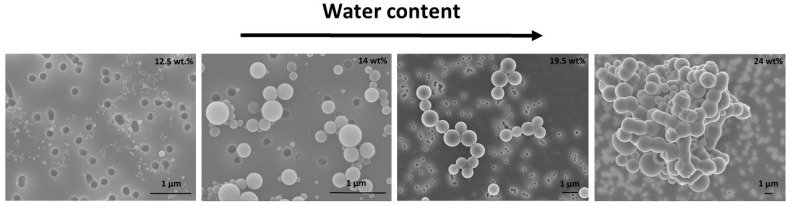
SEM images of mesoscopic PS-b-PAA structures with variation of water content. Spherical micelles (12.5 wt%), vesicles (14 wt% and 19.5 wt%), and large network-like structures (24 wt%).

To establish an efficient encapsulation process, this section will highlight various factors that influence encapsulation. Specifically, various modification types (APTES with comparatively hydrophilic properties and APTES–decanoic acid with highly hydrophobic properties) and particle sizes (20 nm and 45 nm) are discussed. SEM and TEM images provide information about the encapsulation and structure of polymer vesicles as well as insights into the fusion processes of the self-assembled vesicles. At this point, it is important to understand how the structure formation of polymer vesicles basically proceeds (i.e., without the presence of nanoparticles). The block copolymer PS-b-PAA is initially completely dissolved in the organic solvent THF. Addition of the selective solvent water results in energetically unfavorable interactions with the hydrophobic part of the polymer (polystyrene), leading to coiling of the polymer chains. The polymer monomers then self-organize so as to minimize the interfacial area. Depending on the water content, the type and dimension of the morphology can vary, as seen in Figure 8. At a low water content (12.5 wt%), small, spherical micelles are initially present, which then change to the structure of vesicles (14 wt%). With a further increase in water content, the vesicles grow in size (19.5 wt%) and can change again to a different morphology, such as the network-like structure at 24 wt% in Figure 8.

For the encapsulation experiments, we chose a water content of 14 wt% to achieve an adequate size ratio between the silica nanoparticles and the vesicles.

**Figure 9:**
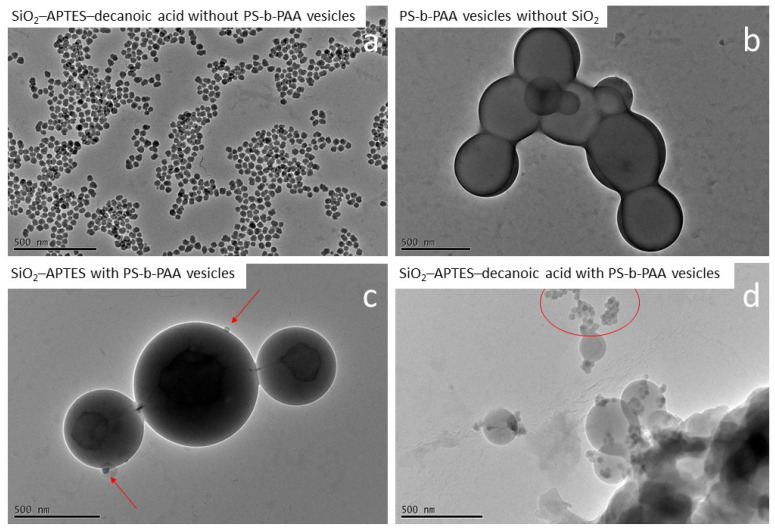
TEM images of the encapsulation experiments of silica nanoparticles in PS-b-PAA vesicles. (**a**) SiO_2_–APTES–decanoic acid nanoparticles (20 nm) without PS-b-PAA vesicles as a reference. (**b**) PS-b-PAA vesicles without SiO_2_ as a reference. (**c**) Encapsulation experiment with SiO_2_–APTES (20 nm). Red arrows indicate silica nanoparticles attached to the vesicle surface. (**d**) Encapsulation experiment with SiO_2_–APTES–decanoic acid (20 nm). Agglomerates of silica nanoparticles outside the vesicles are visible encircled in red.

Figure 9a presents the used silica nanoparticles modified with APTES and decanoic acid for comparison, and Figure 9b presents the formed PS-b-PAA vesicles (480 nm ± 80 nm) in the absence of nanoparticles.

In the presence of APTES-modified silica nanoparticles (mass ratio PS-b-PAA to silica of 10 to 1), the PS-b-PAA structures were present as defined polymer vesicles similar to the reference experiment without silica nanoparticles. Furthermore, these vesicles were partially connected to one another in a chain-like manner (see Figure 9c). It can, therefore, be assumed that no morphology changes occurred due to the presence of APTES-modified silica particles. In terms of encapsulation, the particles attached to the membrane of the vesicles are visible (see red arrows). Furthermore, we assume that some silica particles are encapsulated directly into the vesicles. We believe that the particles bind with the polymer material primarily due to the formation of hydrogen bonds between the amino group of the modified particles and the carboxyl group of the block copolymer. However, the efficiency of the encapsulation does not yet appear to be optimal, as based on the mass ratio of PS-b-PAA to silica of 10 to 1, presumably more particles should have been encapsulated. To increase efficiency, it might be advantageous to use slightly more hydrophobic surface properties. For example, the silica nanoparticles modified with APTES and hexanoic acid might have optimal hydrophobicity.

**Figure 10:**
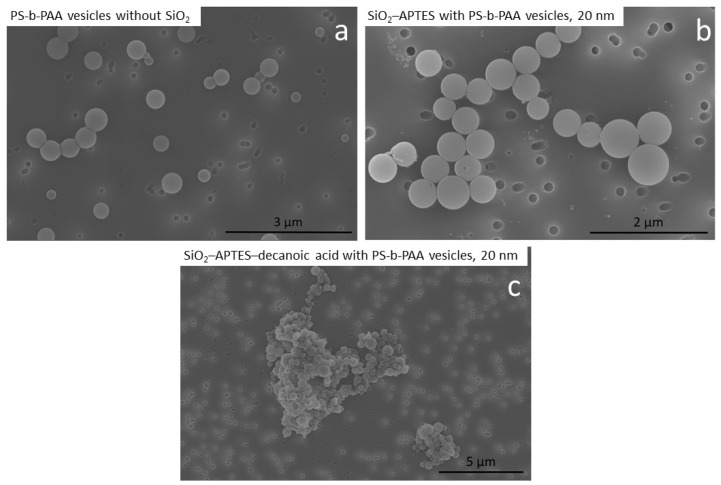
SEM images of the encapsulation experiments of silica nanoparticles in PS-b-PAA vesicles. (**a**) Encapsulation experiment with PS-b-PAA vesicles without SiO_2_ as a reference. (**b**) Encapsulation experiment with SiO_2_–APTES (20 nm). (**c**) Encapsulation experiment with SiO_2_–APTES–decanoic acid (20 nm).

In contrast, we found that in the experiment with APTES–decanoic acid–modified particles, the particles were highly agglomerated and mainly found outside the vesicles while only a few defined vesicles and undefined assemblies were detected (see Figure 9d). Similar results are also visible in the SEM images (compare Figure 10). In the experiments without silica nanoparticles and with APTES-modified particles, defined vesicle structures could be observed (Figure 10a,b), whereas in the experiment with APTES–decanoic acid–modified silica nanoparticles, many large agglomerates were detected (Figure 10c).

This suggests that the silica particles modified with decanoic acid are too hydrophobic for the co-precipitation encapsulation process used here. The addition of water as a selective solvent causes the precipitation of the water-insoluble part of the block copolymer (polystyrene) above a critical amount. However, if the hydrophobic silica particles have a stability limit at a lower water content than polystyrene, the modified particles agglomerate before the vesicles have completely formed, disrupting the self-assembly process and leading to the insufficient encapsulation of the particles as well as increased agglomeration among the vesicles.

**Figure 11:**
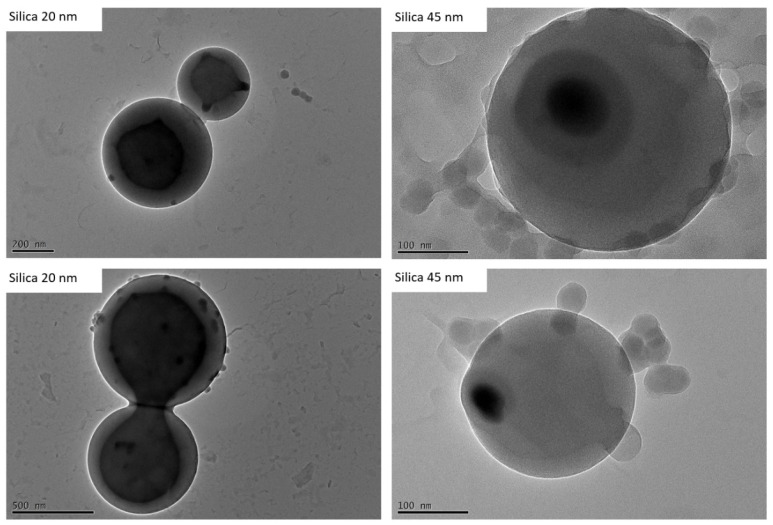
Exemplary TEM images of encapsulation experiments of APTES-modified silica particles with PS-b-PAA vesicles. The image on the **left** shows 20 nm silica particles, and the one on the **right** shows 45 nm silica particles.

We also examined the influence of the particle size of the silica particles on the encapsulation. This was of particular interest with regard to the composite properties of the nanoparticle–polymer hybrids (such as mechanical stability), where a correlation with particle size is likely. Figure 11 shows exemplary TEM images from the experiments with APTES-modified silica particles of 20 nm and 45 nm. We found that defined vesicles can be generated with both particle sizes. The sizes examined here, therefore, do not appear to have any negative influence on the structural formation of the vesicles.

**Figure 12:**
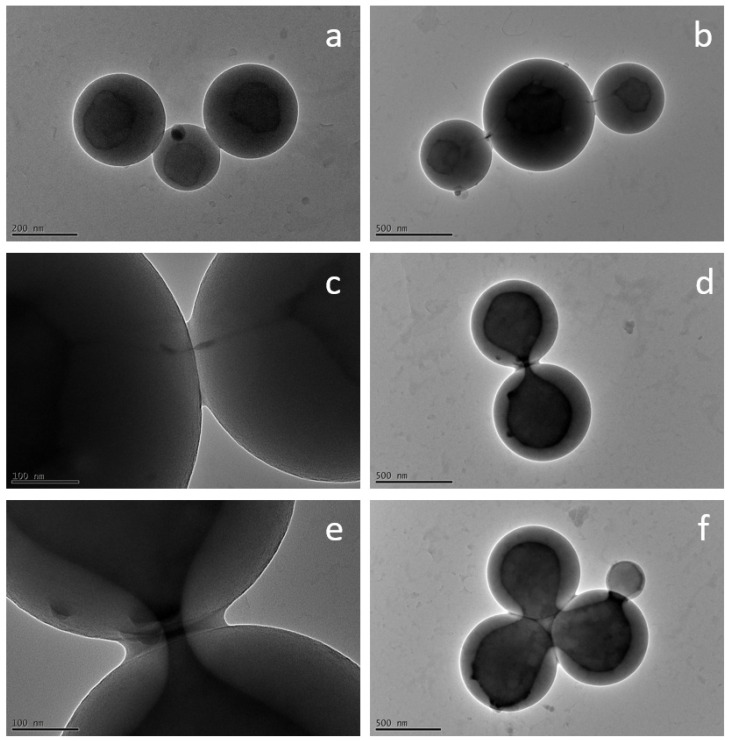
Exemplary TEM images illustrating the fusion process of PS-b-PAA vesicles with APTES-modified silica nanoparticles. (**a**) Docked vesicles (encapsulation attempt with APTES-modified 45 nm silica nanoparticles). (**b**,**c**) Semi-fused intermediates with the formation of fusion pores (encapsulation attempt with APTES-modified 20 nm silica nanoparticles). (**d**–**f**) Opening of the fusion pores (encapsulation attempt with APTES-modified 20 nm silica nanoparticles).

During the encapsulation experiments, we made some interesting observations regarding the fusion behavior of the polymer vesicles. As already mentioned, due to their high molecular weights, block copolymers are suitable for investigating inter- and intra-vesicular reorganization processes, such as those that take place during fusion. Figure 12 shows some exemplary TEM images that show different states during fusion of the self-assembled vesicles, analogous to the basic processes in membrane fusion described in the literature (compare Figure 1). It was not possible to trace the fusion process directly over time, since the duration of the experiments was always the same and therefore, all images show the same point in time. However, there are also different fusion states within a sample, since new fusion processes are continuously initiated and not all vesicles fuse at the same time.

Figure 12a depicts three vesicles that are connected to each other via their outer membrane but still have separate cores. In Figure 12b,c, the cores are seen to be linked by thin filaments, forming a hemifused state, which occurs due to the fusion of the outer membranes and the subsequent creation of a fusion pore (filament). Figure 12d,e displays how this pore continues to open until the interiors of the vesicles are largely fused together (Figure 12f), which can be attributed to further rearrangement mechanisms. A fully fused vesicle could not be observed because the process time of 3 h was apparently not long enough for complete fusion. However, this is likely to occur with passage of time. Interestingly, Qu et al. [28] were able to make the same observations using TEM images, showing vesicle fusion intermediates during the self-assembly process.

We do not assume that the fusion processes were triggered by the high voltage of the TEM, since no significant changes of the polymer vesicles during the duration of a regular measurement could be observed. Furthermore, Qu et al. [28] do not describe any special method for preparing the samples for the TEM measurements. Compared with low-molecular-weight organic amphiphile-based structures, the polymer vesicles are probably more resistant to high voltage due their high molecular weights and their compactness because of their spherical shape.

## 4. Conclusions

In this study, for the first time, the preparation of nanoparticle–polymer hybrids of silica nanoparticles and self-assembled PS-b-PAA vesicles by the co-precipitation method was demonstrated. Important factors influencing the hybrids, such as the particle size and the modification type of the silica nanoparticles, were further elucidated. We found that the nanoparticle size (20 nm and 45 nm) had no negative effects on the structure formation. The modification type, however, had a great strong on the vesicles. When silica nanoparticles were modified with APTES, defined vesicles with encapsulated particles were formed. However, when the silica nanoparticles were modified with APTES and decanoic acid, the vesicles were agglomerated and no encapsulation took place, indicating that hydrophilicity is a crucial factor in encapsulation. In addition, during the experiments with APTES-modified silica nanoparticles, vesicle fusion processes were observed via TEM images, providing a deeper understanding of phase transformation processes.

In the near future, our goal is to focus on a concrete application of nanoparticle–polymer hybrids. Therefore, the field of intrinsically self-healing materials seems appropriate, since supramolecular structures of block copolymers appear promising in the field of self-healing [24,95].

## Data Availability

The data presented in this study are available on request from the corresponding author.
